# Prognostic value of MR-detected mandibular nerve involvement: potential indication for future individual induction chemotherapy in T4 nasopharyngeal carcinoma

**DOI:** 10.1007/s00432-022-04533-w

**Published:** 2023-01-06

**Authors:** Wenjie Huang, Shuqi Li, Chao Luo, Zhiying Liang, Shumin Zhou, Haojiang Li, Yi Cai, Shaobo Liang, Guangying Ruan, Peiqiang Cai, Lizhi Liu

**Affiliations:** 1 Departmentof Radiology, Sun Yat-Sen University Cancer Center, State Key Laboratory of Oncology in South China, Collaborative Innovation Center for Cancer Medicine, Guangdong Key Laboratory of Nasopharyngeal Carcinoma Diagnosis and Therapy, 651 Dongfeng Road East, Guangzhou, 510060 Guangdong People’s Republic of China; 2 Department of Radiology, Shengli Oilfield Central Hospital, No. 31 Jinan Road, Dongying District, Dongying, 257034 Shandong People’s Republic of China; 3 Department of Radiation Oncology, First People’s Hospital of Foshan, Foshan, 528000 Guangdong People’s Republic of China; 4 Department of Radiation Oncology, The Third Affiliated Hospital of Sun Yat-Sen University, Guangzhou, 510000 Guangdong People’s Republic of China

**Keywords:** Induction chemotherapy, Mandibular nerve, Advanced nasopharyngeal carcinoma, Multivariate analysis

## Abstract

**Purpose:**

To investigate the prognostic significance of MR-detected mandibular nerve involvement (MNI) and its value for induction chemotherapy (IC) administration in patients with nasopharyngeal carcinoma (NPC) and T4 disease.

**Methods:**

This retrospective study enrolled 792 non-metastatic, biopsy-proven NPC patients. Univariate and multivariate analysis were used to evaluate potential prognosticators. The inter-observer agreement was assessed by the kappa values.

**Results:**

MR-detected MNI was observed in 141 (72.3%) patients among 195 patients with T4 disease, with excellent agreement between the readers (kappa = 0.926). Patients with MR-detected MNI presented better 5-year overall survival (OS) (hazard ratio [HR], 0.40; *P* = 0.006) than those with MR-negative MNI. Of these patients, IC treatment was verified as an independent factor (HR: 0.35; *P* = 0.014) with preferable effect on OS.

**Conclusion:**

MR-detected MNI could serve as an independent favorable prognostic predictor for OS in NPC patients with stage T4, which should be considered for stratifying these patients for IC administration.

## Introduction

Nasopharyngeal carcinoma (NPC) is a malignancy originating from nasopharyngeal mucosa, accounting for 129,079 new cases and 72,987 deaths in 2018 (Bray et al. 2018). According to the 8th edition of the American Joint Committee on Cancer (AJCC) staging system, intracranial extension, cranial nerve (CN) invasion, involvement of hypopharynx, orbit, parotid gland, and/ or extensive soft tissue infiltration beyond the lateral surface of the lateral pterygoid muscle in patients with NPC are classified as T4 stage and associated with poor prognosis (Amin et al. 2016). Inevitably, induction chemotherapy (IC) is applied to these advanced patients for better tumor control and survival (Sun et al. 2016; Li et al. 2019). The toxicity of IC should not be overlooked as adverse events of grade 4 in the IC group were reported in 18% of locoregionally advanced patients (Sun et al. 2016), compared with 1% in the standard group who received chemoradiotherapy only. Many studies have attempted to find the imaging marker that could divide T4 patients into groups with distinct prognoses and guide decision-making (Hu et al. 2011; Chen et al. 2012; Cao et al. 2017; Hung et al. 2014; Liao et al. 2016). Nevertheless, prognostic heterogeneity of T4 patients is still a clinical issue in that not all patients would benefit from IC, and no substantial evidence currently supports the correlation between the benefits of IC use and known imaging markers in T4 stage.

Clinical staging and pathologic variables are the main prognostic determinants and function as the basis for appropriate therapy selection at the time of diagnosis. As such, perineural invasion (Bakst et al. 2019; Biau et al. 2019), characterized by microscopic CN invasion and spread along nerve sheath, is widely accepted as a pathologic marker associated with high incidence of disease recurrence and distant metastasis. Unlike the type defined by histological confirmation, the other category of CN invasion is macroscopic perineural spread, which is a radiological or clinical finding on larger nerves. Among head and neck tumors, the trigeminal nerve is the most vulnerable structure to invasion, and its branches penetrates the skull base foramina, respectively, providing a potential channel for tumor spread and dissemination (Biau et al. 2019; Bathla and Hegde 2013; Cui et al. 2009). As the prominent branch of the trigeminal nerve, the mandibular nerve is surrounded by rich vessels and revealed as the most frequent CN invasion in patients with NPC (Su and Lui 1996; Liu et al. 2009). Liang et al. (2009) summarized infiltration routes to cavernous sinus in NPC patients and pointed out that the foramen ovale, where mandibular nerve passes, is the most common privileged infiltration. As a platform where the invasive intra- and extra-cranial tumor communicates, mandibular nerve involvement (MNI) is distinguishable; however, its value on prognosis and decision-making is unclear and is often ignored as a part of extensive T4 infiltration.

Magnetic resonance (MR) has a great sensitivity for evaluating perineural tumor spread and can detect CN invasion with better accuracy than that clinical evaluation (Bakst et al. 2019). Some studies reported that the radiological evaluations of perineural spread have good associations with pathological results (Williams et al. 2003; Gandhi et al. 2011). Therefore, we aimed to verify the prognostic value of MR-detected MNI in T4 patients and attempted to verify whether this subset of patients could potentially benefit from the available IC currently used.

## Materials and methods

### Patients

Retrospectively, we enrolled 3814 patients with biopsy-confirmed NPC from January 2010 to January 2013 in hospital. The inclusion criteria are: (1) diagnosed as NPC with pathological examination; (2) completed medical records including pretreatment MR examination, Epstein–Barr virus (EBV) DNA load measurements and treatment records; (3) underwent intensity-modulated radiotherapy (IMRT). The exclusion criteria are: (1) presented with distant metastasis at first diagnosis (*N* = 24); (2) had concurrent tumor at other parts of body (*N* = 20). (3) with incomplete MR images for head and neck regions (*N* = 5); (4) with incomplete clinical data (*N* = 2973). At last, a total of 792 patients were enrolled in our study. Of these, 195 patients were diagnosed with stage T4 disease without any metastasis or concurrent malignancies. All pretreatment examinations were done as previously described (Liu et al. 2020). An entire body bone scan or positron emission tomography/computed tomography was performed when clinicians suspected distant metastasis. The serum EBV-DNA load measurements were done as previously described (Shao et al. 2004). This study was conducted in accordance with the 1964 Helsinki Declaration and approved by committees of the Institutional Review Board in our center. As this study was a retrospective study, informed patient consent was waived.

### MR protocol

All patients underwent MR examinations with a 1.5-T system (Signa CV/i, General Electric Healthcare) or 3.0-T system (Magnetom Tim Trio, Siemens). Scanning region is from the suprasellar cistern to the superior margin of the thoracic cage. Before enhancement, sequence included fast spin-echo (FSE) T1-weighted images (T1WI) in axial, coronal, and sagittal planes (TR = 540 ms, TE = 11.8 ms) and FSE T2-weighted images (T2WI) in axial planes (TR = 4000 ms, TE = 99 ms). Enhanced images included T1WI axial and sagittal sequences and fat-suppressed T1WI coronal sequences. Section thickness of axial, sagittal and coronal planes was 5 mm, 3 mm and 2 mm, respectively, with the section gaps of 1 mm for axial and sagittal planes and 0.5 mm for coronal planes.

### Imaging assessment and criteria for MR-detected MNI

Two experienced radiologists with more than 10-year experiences in NPC diagnosis reviewed randomly selected MR images for 51.3% (100/195) of the cases before separately evaluating the remaining MR images. Disagreements were settled through group discussions. The diagnostic criteria of T4 NPC were based on the 8th edition of AJCC staging system (Amin et al. 2016).

**Fig. 1:**
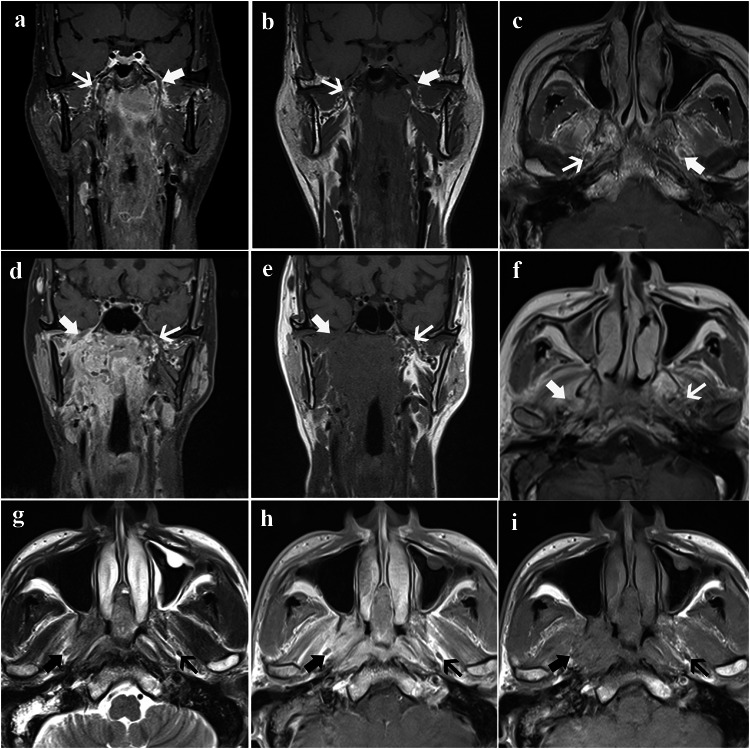
Magnetic resonance images in three cases with mandibular nerve involvement. Case 1: **a** Enhanced coronal, **b** coronal, and **c** axial T1-weighted images in a 47-year-old man show the enlargement of the mandibular nerve (thick arrow) and normal mandibular nerve (thin arrow). Case 2: **d** Enhanced coronal, **e** coronal, and **f** axial T1-weighted images in a 77-year-old female show the enlargement and abnormal enhancement of the mandibular nerve (thick arrow) and normal mandibular nerve (thin arrow). Case 3: **g** Axial T2-weighted image, **h** axial, and **i** enhanced axial T1-weighted images in a 58-year-old man show the enlargement of the mandibular nerve (thick arrow) and normal mandibular nerve (thin arrow). In all three patients, abnormal enhancement of the soft tissue along mandibular nerve was observed

The mandibular nerve originates from trigeminal ganglion, bypasses the cavernous sinus, and distributes between medial pterygoid muscle and lateral pterygoid muscle. The mandibular nerve mainly contains the basicranial segment and extra-cranial segment. We defined MR-detected MNI as extra-cranial segment (i.e., segment in masticator space) involvement and/or basicranial segment (segment through the foramen oval) involvement based on MR. The diagnostic criteria for MR-detected MNI were as follows (Bakst et al. 2019; Biau et al. 2019; Bathla and Hegde 2013; Badger and Aygun 2017): (i) asymmetric enlargement seen in MNI accompanied by heterogeneous enhancement in contrast enhancement coronal T1WI with fat-suppressed; (ii) irregular soft tissue enhancement replacing fat space in the pathway of CN; asymmetrical enlargement can also be seen in the affected foramen oval (Fig. 1).

We defined those patients with masticatory muscles involvement as the MMI and defined those patients with cavernous sinus involvement as CSI.

### Treatment

The target volumes in all NPC patients were treated using IMRT, and were delineated following an individualized delineation protocol (Zhao et al. 2004), according to the International Commission on Radiation Units and Measurements Reports 50 and 62 (Li et al. 2015) as described (Lai et al. 2011). The disease stages were reassessed for all patients using the 8th edition of the AJCC staging system. In total, 195 (24.6%) of the 792 patients were diagnosed with stage T4 disease. Of these patients, 138 (70.8%) received IC and 57 (29.2%) did not receive IC; 157 (80.5%) patients received concurrent chemotherapy and 38 (19.5%) did not. All patients received IMRT and adjuvant chemotherapy. Surgery, reirradiation, and chemotherapy were the choices for salvage treatment in cases with recurrent and refractory diseases. Our chemotherapy regimen was based on cisplatin, with gemcitabine or docetaxel or a combination of both. The regimens of IC and concurrent chemotherapy are as mentioned previously (Liu et al. 2020).

### Follow-up

Within the 5-year of the follow-up period, patients were evaluated using MR and clinical examination every three months during the first two years, and every six months thereafter. The main survival outcome was OS, a period from the date of initial treatment to the date of death from any cause or the last follow-up. Distant metastasis-free survival (DMFS), locoregional-free survival (LRFS), and progression-free survival (PFS) were the secondary survival outcomes, which defined as the time from date of initial treatment to the date of relevant events or the last follow-up.

### Statistical analysis

Bilateral confidence intervals were used to assess the variables. Kappa values were calculated to evaluate the reliability of the inter-observer agreement on different structure reading, including the segment of extra-cranial mandibular nerve, the segment of basicranial mandibular, mandibular nerve, and cavernous sinus. Univariate and multivariate analyses were used to evaluate the prognostic value of MR-detected MNI. After univariate analyses, variables with *P*-values less than 0.05 were selected in the multivariate Cox proportional hazards model that was used to estimate the likelihood of hazard ratios (HRs) and their 95% confidence intervals (CIs). Actual survival rates were determined using the Kaplan–Meier method, and difference was compared using log-rank test. Considering the influence from surrounding important structures, including masticatory muscles or cavernous sinus, we conducted subgroup analysis to compare which involved structure has the superior prognostic value. Thus, we observed the prognosis of MR-detected MNI in subgroup of T4 patients with MMI or CSI, respectively. All statistical analyses were performed using Software R-version 3.2.5 (https://www.r-project.org/). Significance was set at *α* = 0.05.

## Results

### Patient characteristics and treatment outcomes

Of 195 (24.6%) patients diagnosed with stage T4 disease, 72.3% (141/195) presented MNI on MR. The patient characteristics are presented in Table 1. The median follow-up period for the entire cohort was 60.3 months (range 1.4–83.4). At the end of the follow-up, 41 patients (21.0%) died, 37 (20.0%) developed distant metastases, 29 (14.9%) developed local regional recurrences, and in 62 (31.8%) the disease progressed. The 5-year OS, DMFS, LRFS, and PFS were 78.1%, 79.9%, 83.4%, and 67.0%, respectively. In terms of reliability of MNI, the kappa value was 0.930, 0.900, 0.926 0,880 for the segment of extra-cranial mandibular nerve, segment of basicranial mandibular nerve, mandibular nerve, cavernous sinus, which indicated almost excellent agreement between the readers (Table 2).

**Table 1:** Clinicodemographic characteristics of the patients with T4 NPC (*n* = 195) and univariate analysis of variables associated with OS

Variables	*n* (%)	OS	
		5 years (%)	Univariate Cox regression *P* value
Age (years)			0.006
13–75	195 (100.0%)	78.1	
Sex			0.488
Male	140 (71.8%)	76.0	
Female	55 (28.2%)	83.2	
KPS			0.674
80	12 (6.2%)	72.7	
90	181 (92.8%)	78.2	
100	2 (1.0%)	100.0	
Symptomatic CN invasion			0.018
No	153 (78.5%)	81.9	
Yes	42 (21.5%)	63.7	
Histologic type			0.887
I	0 (0%)	NA	
II	9 (4.6%)	75.0	
III	186 (95.4%)	78.2	
EBV (1000 copies/ml)			0.907
< 1	58 (29.7%)	79.1	
< 10	46 (23.6%)	75.6	
≥ 10	91 (46.7%)	78.6	
N classification			0.185
N0	27 (13.8%)	81.5	
N1	122 (62.6%)	78.5	
N2	33 (16.9%)	80.7	
N3	13 (6.7%)	58.7	
IC			0.237
No	57 (29.2%)	72.4	
Yes	138 (70.8%)	80.4	
CSI			0.977
No	87 (44.6%)	78.6	
Yes	108 (55.4%)	77.2	
MMI			0.058
No	96 (49.2%)	83.7	
Yes	99 (50.8%)	72.3	
MNI			0.019
No	54 (27.7%)	67.0	
Yes	141 (72.3%)	82.3	

*OS* overall survival, *CN* cranial nerve, *EBV* Epstein–Barr virus, *IC* induction chemotherapy, *CSI* cavernous sinus involvement, *MMI* masticatory muscles involvement, *MNI* mandibular nerve involvement, *NPC* nasopharyngeal carcinoma, *KPS* Karnofsky Performance Status, *NA* not available

**Table 2:** Interobserver variability for variables evaluated in this study (*n* = 100)

	Observer1	Observer2	Disagreement	Kappa
Segment of extra-cranial mandibular nerve				0.930
No	31	32	2	
Yes	69	68	1	
Segment of basicranial mandibular nerve				0.900
No	48	47	2	
Yes	52	53	3	
Mandibular nerve				0.926
No	29	28	2	
Yes	71	72	1	
Cavernous sinus				0.880
No	49	47	2	
Yes	51	53	4	

### Prognostic value of MR-detected MNI in T4 patients

**Fig. 2:**
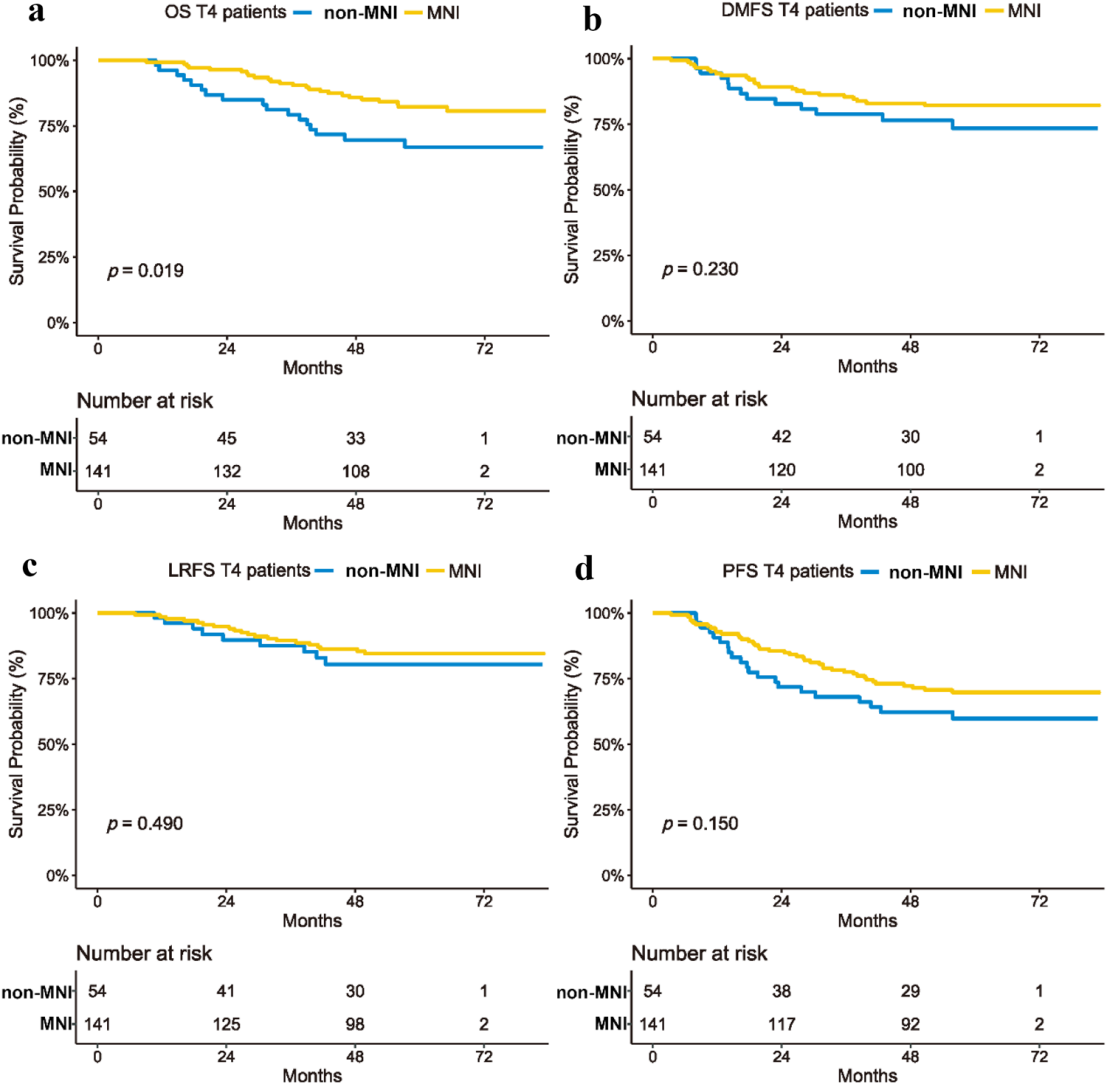
Kaplan–Meier’s curves of the 5-year OS, DMFS, LRFS and PFS in T4 NPC patients with and without MNI. **a** The survival curve in T4 NPC patients with and without MNI was well separated in terms of OS. **b**–**d** The survival curve in T4 NPC patients with MNI was superior to that in patients without MNI in terms of DMFS, LRFS, and PFS; however, no significant difference was observed. *OS* overall survival, *DMFS* distant metastasis-free survival, *LRFS* locoregional-free survival, *PFS* progression-free survival, *MNI* mandibular nerve involvement

In the univariate analysis (Table 1), the 5-year OS was significantly different between stage T4 patients with and without MR-detected MNI (82.3% vs. 67.0%, *P* = 0.006). Further, those with MR-detected MNI showed slightly better DMFS (82.1% vs. 73.5%, *P* = 0.230), LRFS (84.5% vs. 80.5%, *P* = 0.490), and PFS (69.8% vs. 59.8, *P* = 0.150) (Fig. 2), although these differences were not significant.

The confounding factors were age, symptomatic CN invasion, and MMI. The prognostic value of MMI approached statistical significance in univariate analysis (*P* = 0.058); thus, we included it in the next multivariate analysis with an interest to compared it with that of MNI and determine which one has superior prognostic value. In the multivariate Cox regression analysis, MR-detected MNI could independently predict 5-year OS (HR = 0.40; 95% CI = 0.21–0.76, *P* = 0.006) in T4 patients (Table 3).

**Table 3:** Multivariate analysis of prognostic factors in patients with T4 NPC

Endpoint	Variables	HR	95% CI	*P* value
OS	MR-detected MNI	0.40	0.21–0.76	0.006
	Age	1.03	1.00–1.05	0.037
	Symptomatic CN invasion	1.97	1.02–3.81	0.044
	MMI	1.94	0.99–3.79	0.054

CI, confidence interval; CN, cranial nerve; HR, hazard ratio; MMI, masticatory muscles involvement; NPC, nasopharyngeal carcinoma; OS, overall survival; MNI, mandibular nerve involvement

Multivariable Cox regression models were adjusted for age, symptomatic CN invasion, and MMI. Although MMI (*P* = 0.058) was not a significant factor in the univariate analysis, we included this as an interested variable based on the report by Chen et al. (Chen et al. 2012). Each subgroup without corresponding variables was set as the reference group, and relative HRs and *P*-values were calculated from the reference group (e.g., the HR of MR-detected MNI for OS was calculated with the subgroup of T4 patients without MR-detected MNI as the reference)

### Subgroup analysis

MMI was observed in 50.8% (99/195) and CSI was observed in 55.4% (108/195) of patients with stage T4 NPC on MR. In the univariate analysis, MMI (*P* = 0.058) and CSI (*P* = 0.997) were not significant prognostic factors (Table 1).

**Fig. 3:**
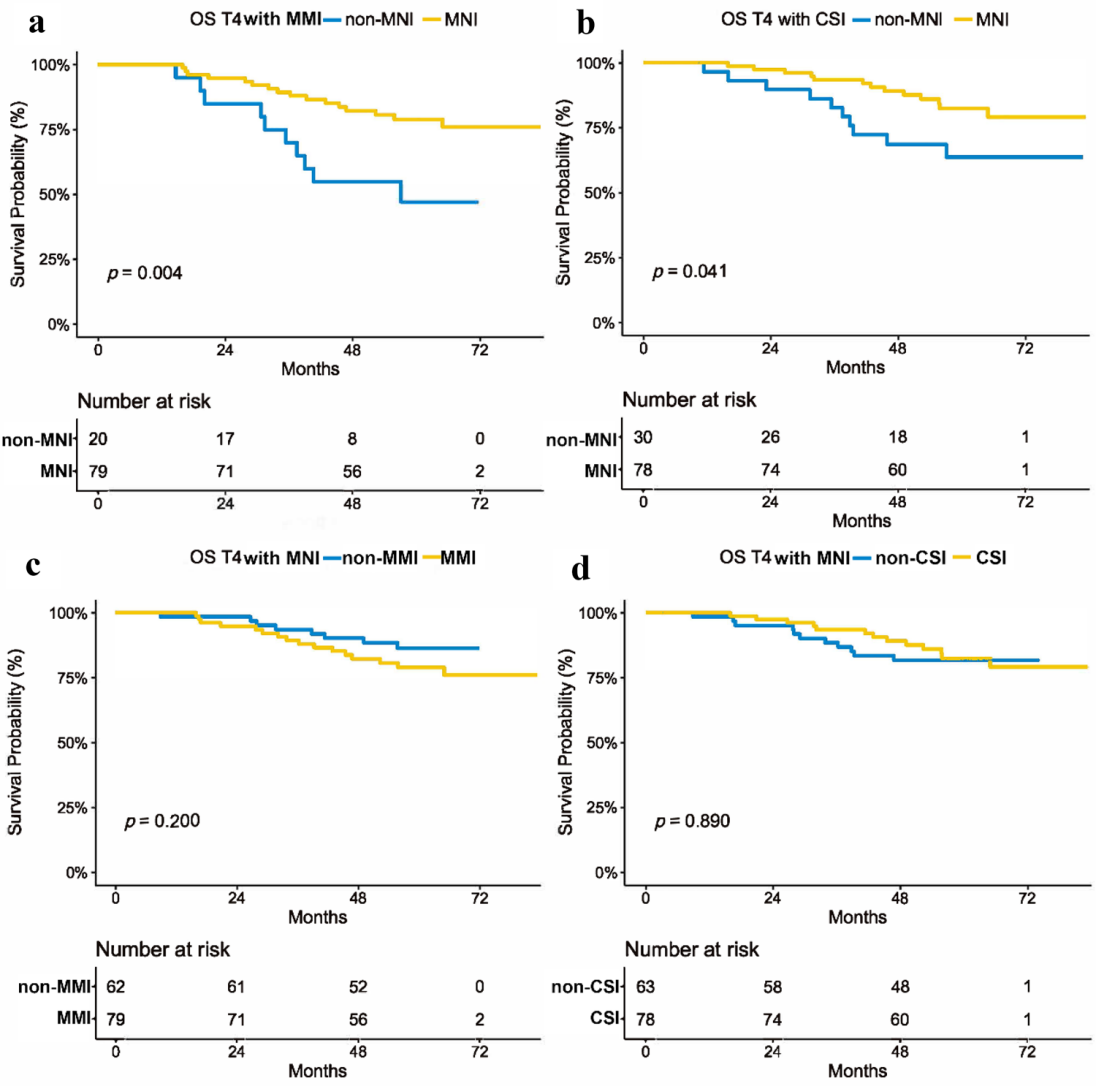
Kaplan–Meier survival curves of subgroup analysis. The OS in patients with MNI was significantly better than that in patients without MNI in subset of T4 patients with MMI (**a**) or CSI (**b**). Conversely, among the T4 NPC patients with MNI, no significant difference was observed between those with or without MMI (**c**) or CSI (**d**). *OS* overall survival, *IC* induction chemotherapy, *MNI* mandibular nerve involvement, *CSI* cavernous sinus involvement, *MMI* masticatory muscles involvement

In subgroup analyses, of T4 NPC patients with MMI, the 5-year OS in those with MNI was higher than that in patients without MNI (*P* = 0.004). Similar results were observed in T4 NPC patients with CSI that MNI patients had better 5-year OS compared to that of patients without MNI (*P* = 0.041). Conversely, among the T4 NPC patients with MNI, no significant difference was observed between those with or without MMI (*P* = 0.200) or CSI (*P* = 0.890), respectively (Fig. 3). MNI could further divide the MMI or CSI patients into distinct prognoses, while those patients couldn’t stratify patients with MNI. MNI could predict OS, independent of the influence of the invasion of masticatory muscles and cavernous sinus.

### Prognostic impact of IC in patients with T4 NPC and MR-detected MNI

Chi-square test showed no significant difference in the distribution between patients with stage T4 disease with and without MR-detected MNI in terms of clinical characteristics (age, sex, Karnofsky Performance Status, histologic type, symptomatic CN invasion, and IC use), tumor burden (EBV-DNA level, tumor volume, and N classification), invasion pattern (the presence of invasion of masticatory muscles, cavernous sinus, foramen rotundum, jugular foramen, hypoglossal canal, superior/inferior orbital tissue/orbital apex, and pterygopalatine fossa), and other CN invasion (trigeminal ganglion, CN II–VI, IX, and XII) (Table 4) except for trigeminal ganglion invasion, hypoglossal canal invasion, and MMI. The presence of ganglion invasion and hypoglossal canal invasion, as well as abovementioned MMI, had no effect on the prognosis of T4 patients. More details of the prognostic significance of different T4 structures invasion are shown in Table 5.

**Table 4:** Chi-square test results in patients with T4 NPC with or without MR-detected MNI

	Variables	Non-MNI (*n* = 54)	MNI (*n* = 141)	*P* value
Tumor burden	EBV (1000 copies/ml)			0.710
	< 1	18 (33.3%)	40 (28.4%)	
	< 10	11 (20.4%)	35 (24.8%)	
	≥ 10	25 (46.3%)	66 (46.8%)	
	Tumor volume^*^			0.985
		70.4 (36.2%)	70.3 (39.1%)	
	N classification			1.000
	N0/1	41 (75.9%)	108 (76.6%)	
	N2/3	13 (24.1%)	33 (23.4%)	
Clinical characteristics	Age (years)			0.097
	13–75	45.7 (13.9)	49.1 (12.0)	
	Sex			0.859
	Male	38 (70.4%)	102 (72.3%)	
	Female	16 (29.6%)	39 (27.7%)	
	KPS			0.318
	80	5 (9.3%)	7 (5%)	
	90	49 (90.7%)	132 (93.6%)	
	100	0 (0%)	2 (1.4%)	
	Symptomatic CN invasion			0.849
	No	43 (79.6%)	110 (78%)	
	Yes	11 (20.4%)	31 (22%)	
	Histologic type			0.259
	II	4 (7.4%)	5 (3.5%)	
	III	50 (92.6%)	136 (96.5%)	
	IC			0.598
	No	14 (25.9%)	43 (30.5%)	
	Yes	40 (74.1%)	98 (69.5%)	
Invasion pattern	CSI			1.000
	No	24 (44.4%)	63 (44.7%)	
	Yes	30 (55.6%)	78 (55.3%)	
	MMI			0.021
	No	34 (63%)	62 (44%)	
	Yes	20 (37%)	79 (56%)	
	Foramen rotundum			0.384
	No	40 (74.1%)	95 (67.4%)	
	Yes	14 (25.9%)	46 (32.6%)	
	Jugular foramen			0.401
	No	51 (94.4%)	126 (89.4%)	
	Yes	3 (5.6%)	15 (10.6%)	
	Hypoglossal canal			0.001
		45 (83.3%)	80 (56.7%)	
		9 (16.7%)	61 (43.3%)	
	Superior/inferior orbital tissue/orbital apex			1.000
	No	46 (85.2%)	120 (85.1%)	
	Yes	8 (14.8%)	21 (14.9%)	
	Pterygopalatine fossa			0.327
	No	28 (51.9%)	61 (43.3%)	
	Yes	26 (48.1%)	80 (56.7%)	
Other CN invasion	Optic nerve			1.000
	No	54 (100.0%)	140 (99.3%)	
	Yes	0 (0)	1 (0.7%)	
	Cavernous sinus segment of III			0.146
	No	51 (94.4%)	123 (87.2%)	
	Yes	3 (5.6%)	18 (12.8%)	
	Cavernous sinus segment of IV			0.102
	No	48 (88.9%)	111 (78.7%)	
	Yes	6 (11.1%)	30 (21.3%)	
	Cavernous sinus segment of V1			0.342
	No	38 (70.4%)	89 (63.1%)	
	Yes	16 (29.6%)	52 (36.9%)	
	Cavernous sinus segment of V2			0.192
	No	38 (70.4%)	85 (60.3%)	
	Yes	16 (29.6%)	56 (39.7%)	
	Cavernous sinus segment of VI			0.091
	No	42 (77.8%)	92 (65.2%)	
	Yes	12 (22.2%)	49 (34.8%)	
	Trigeminal ganglion			0.017
	No	40 (74.1%)	78 (55.3%)	
	Yes	14 (25.9%)	63 (44.7%)	
	Cistern segment of V			0.166
	No	54 (100.%)	133 (94.3%)	
	Yes	0 (0)	8 (5.7%)	
	Cistern segment of IX			1.000
	No	53 (98.1%)	139 (98.6%)	
	Yes	1 (1.9%)	2 (1.4%)	
	Cistern segment of XII			0.618
	No	52 (96.3%)	138 (97.9%)	
	Yes	2 (3.7%)	3 (2.1%)	

*OS* overall survival, *CN* cranial nerve, *EBV* Epstein–Barr virus, *IC* induction chemotherapy, *CSI* cavernous sinus involvement, *MMI* masticatory muscles involvement, *MNI* mandibular nerve involvement, *KPS* Karnofsky Performance Status

^*^Tumor volume (from GTV) 10.0–197.7 cm^3^

**Table 5:** Univariate analysis of different T4 structures associated with the survival outcomes (n = 195 patients)

Variables	*n* (%)	OS		DMFS		LRFS		PFS	
		5 years	*P *value	5 years	*P *value	5 years	*P *value	5 years	*P *value
Cavernous sinus			0.977		0.806		0.549		0.701
No	87 (44.6%)	78.6		79.6		86.1		69.5	
Yes	108 (55.4%)	77.2		79.8		81.4		64.8	
Prepontine cistern			0.677		0.654		0.697		0.559
No	194 (99.5%)	78.0		79.8		83.4		66.9	
Yes	1 (0.5%)	NA		NA		NA		NA	
Brain stem			0.608		0.635		0.670		0.526
No	194 (99.5%)	77.9		79.8		83.4		66.9	
Yes	1 (0.5%)	100		100		100		100	
Temporalis			0.618		0.635		0.670		0.526
No	194 (99.5%)	77.9		79.8		83.4		66.9	
Yes	1 (0.5%)	100		100		100		100	
Masseter			0.473		0.501		0.545		0.369
No	193 (99%)	77.8		79.7		83.3		66.7	
Yes	2 (1%)	100		100		100		100	
Parotid space			0.492		0.513		0.019		0.489
No	188 (96.4%)	78.4		80.2		84.5		67.5	
Yes	7 (3.6%)	71.4		71.4		53.6		53.6	
Posterior maxillary space			0.745		0.139		0.836		0.995
No	173 (88.7%)	77.9		81.7		83.3		67.1	
Yes	22 (11.3%)	79.1		66.0		84.5		66.1	
Optic chiasm			0.608		0.635		0.670		0.526
No	194 (99.5%)	77.9		79.8		83.4		66.9	
Yes	1 (0.5%)	100		100		100		100	
Optic nerve			0.608		0.635		0.670		0.526
No	194 (99.5%)	77.9		79.8		83.4		66.9	
Yes	1 (0.5%)	100		100		100		100	
Inferior/superior orbital fissure/orbital apex			0.417		0.833		0.193		0.887
No	166 (85.1%)	79.7		80.2		81.9		67.0	
Yes	29 (14.9%)	68.1		77.6		92.0		67.0	
Cavernous sinus segment of III			0.691		0.233		0.560		0.783
No	174 (89.2%)	78.9		81.1		84.1		67.4	
Yes	21 (10.8%)	67.8		69.6		77.6		64.2	
Cavernous sinus segment of IV			0.539		0.363		0.812		0.891
No	159 (81.5%)	79.5		81.3		83.3		67.4	
Yes	36 (18.5%)	70.9		73.7		83.6		65.0	
Cavernous sinus segment of V1			0.332		0.484		0.367		0.807
No	127 (65.1%)	80.4		81.7		81.8		67.0	
Yes	68 (34.9%)	73.2		76.4		86.1		66.9	
Cavernous sinus segment of V2			0.526		0.698		0.440		0.707
No	123 (63.1%)	79.7		81.1		82.1		66.7	
Yes	72 (36.9%)	74.8		77.8		85.4		67.3	
Cavernous sinus segment of VI			0.109		0.617		0.998		0.871
No	134 (68.7%)	81.5		81.1		83.7		68.0	
Yes	61 (31.3%)	69.8		77.0		82.6		64.6	
Trigeminal nerve in cistern			0.629		0.734		0.271		0.811
No	187 (95.9%)	79.6		79.6		82.8		66.9	
Yes	8 (4.1%)	75.6		87.5		100		72.9	
Glossopharyngeal in cistern			0.384		0.411		0.458		0.273
No	192 (98.5%)	77.7		79.6		83.2		66.5	
Yes	3 (1.5%)	100		100		100		100	
Hypoglossal nerve in cistern			0.981		0.928		0.674		0.671
No	190 (97.4%)	78.0		79.9		83.6		67.2	
Yes	5 (2.6%)	80.0		80.0		75.0		60.0	
Trigeminal ganglion			0.629		0.964		0.749		0.981
No	118 (60.5%)	79.6		80.2		83.1		67.7	
Yes	77 (39.5%)	75.6		79.4		83.8		65.9	
Symptomatic CN invasion			0.018		0.021		0.390		0.069
No	153 (78.5%)	81.9		83.5		82.1		70.3	
Yes	42 (21.5%)	63.7		66.1		89.0		54.5	
Foramen ovale			0.059		0.767		0.843		0.298
No	81 (41.5%)	71.36		78.3		83.1		63.0	
Yes	114 (58.5%)	82.79		80.8		83.7		69.9	
Foramen lacerum			0.852		0.844		0.549		0.728
No	50 (25.6%)	79.6		81.8		86.3		69.6	
Yes	145 (74.4%)	77.5		79.1		82.4		66.1	
Foramen rotundum			0.344		0.284		0.953		0.452
No	135 (69.2%)	80.5		81.8		83.8		68.6	
Yes	60 (30.8%)	72.4		75.6		82.4		63.4	

*OS* overall survival, *DMFS* distant metastasis-free survival, *LRFS* locoregional-free survival, *PFS* progression-free survival, *NA* not available

**Fig. 4:**
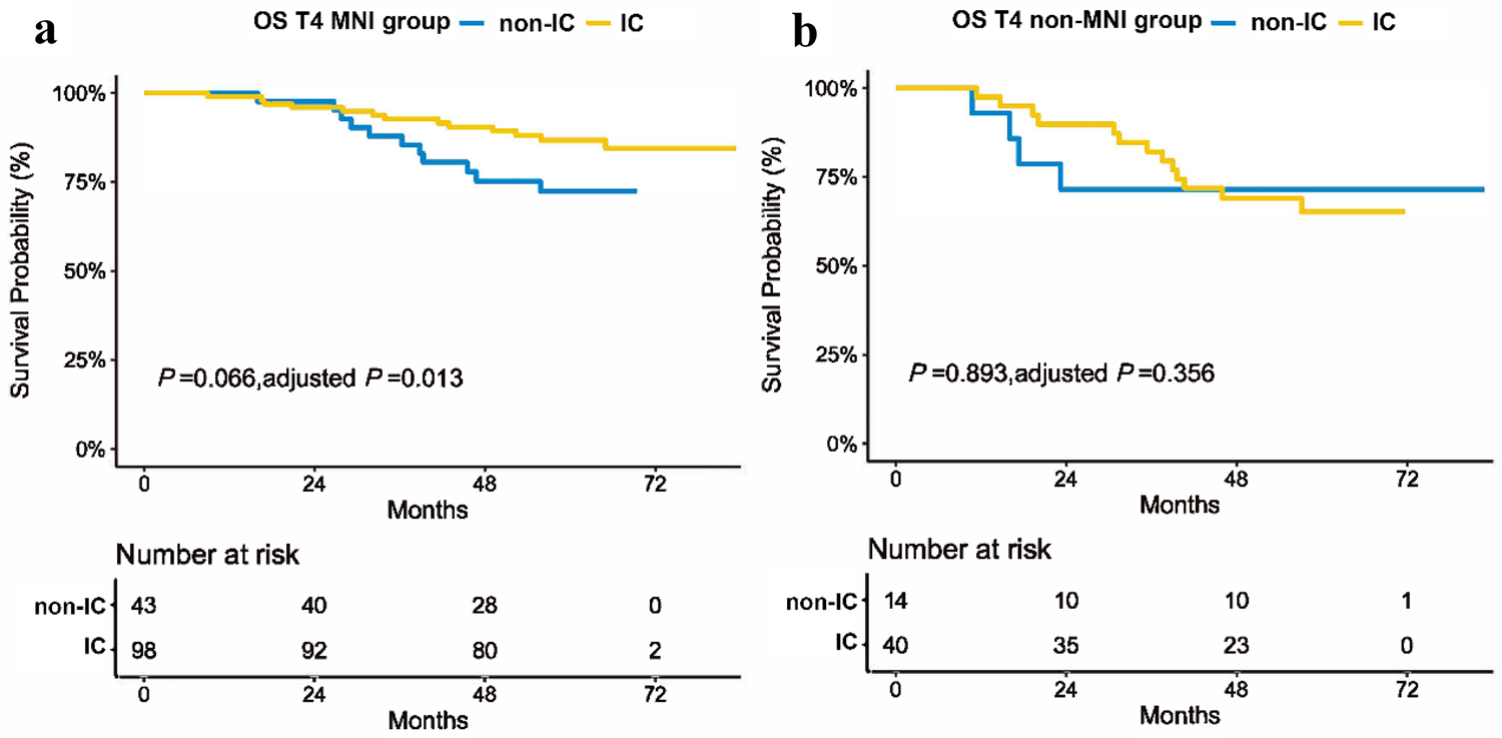
Kaplan–Meier survival curves in T4 MNI patients with and without IC. **a** After adjusting age, symptomatic CN invasion, and MMI, IC significantly improved the OS of the T4 NPC patients with MNI. **b** In T4 patients without MNI, no significant difference was observed between patients who received IC and those who did not receive IC. *OS* overall survival, *IC* induction chemotherapy, *MNI* mandibular nerve involvement

Stepwise, we evaluated the significance of IC use in T4 patients. We stratified the 195 patients with stage T4 disease into two groups: those with MNI group and those with non-MNI group. Using the same confounding variables as mentioned above, Kaplan–Meier and adjusted Kaplan–Meier analyses were performed to verify the prognostic value of IC in patients with and without MNI, respectively (Fig. 4).

**Fig. 5:**
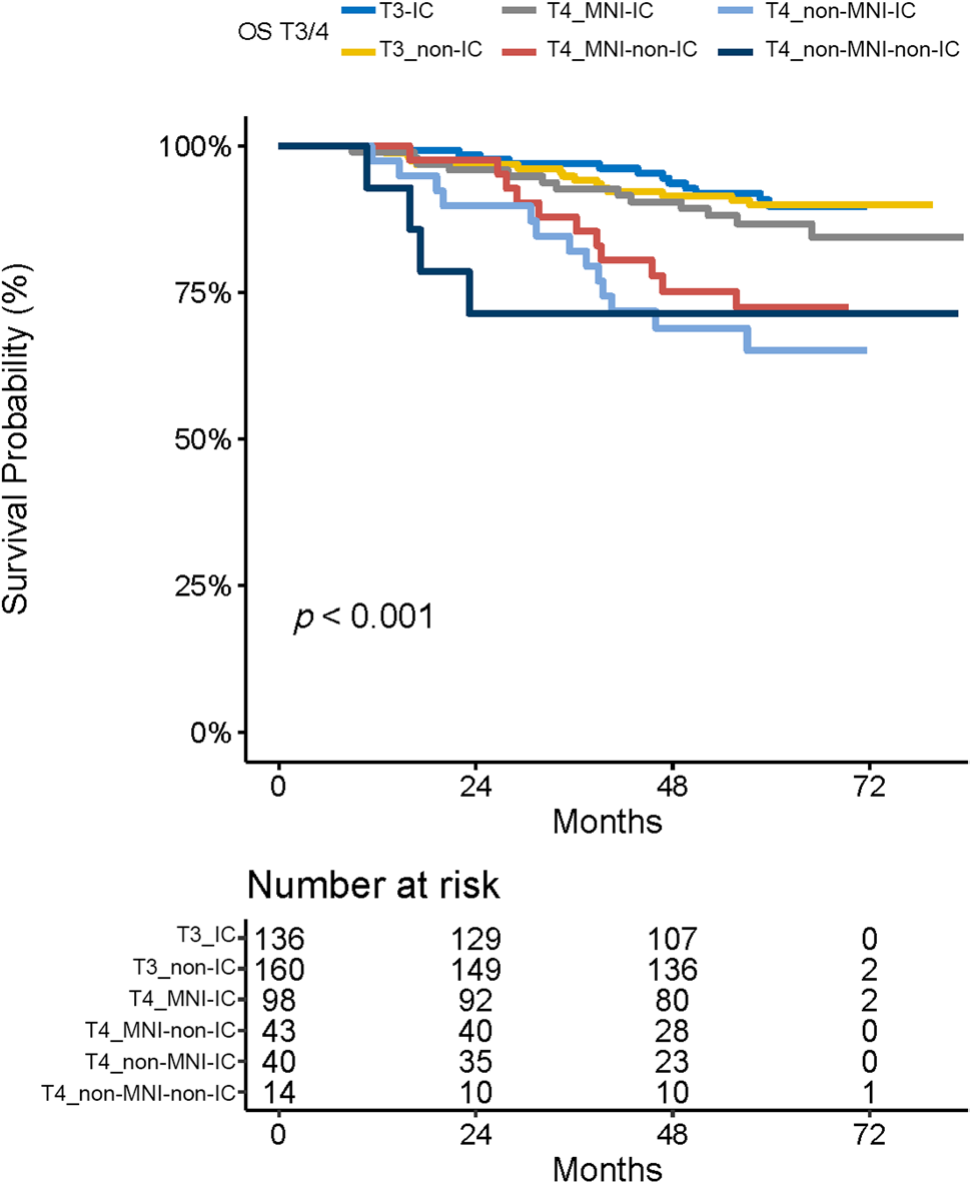
The OS in T3 NPC patients and T4 NPC patients with and without MNI. Regardless of the IC used, there were no significant differences of OS in T3 or T4 non-MNI groups. The OS in T4 MNI group, if received IC, was approaching to that in T3 patients; if not received IC, it was similar to that in T4 non-MNI group. *OS* overall survival, *IC* induction chemotherapy, *MNI* mandibular nerve involvement

In the MNI group, IC was an independent favorable factor for OS (HR = 0.35, 95% CI = 0.15–0.80, *P* = 0.014) (Table 6). In the non-MNI group, no significant difference was observed in the 5-year OS regardless of IC administration (*P* = 0.893). Moreover, without IC administration, the prognosis of MNI group is as worse as the non-MNI group. With IC administration, 5-year OS in the MNI group was close to that observed in patients with stage T3 NPC (86.7% vs. 89.9%, *P* = 0.242) in spite of the fact that there was no significant difference in OS among T3 patients with or without IC administration (*P* = 0.893) (Fig. 5). Furthermore, there was no significant difference in the distribution of different variables, including age, Karnofsky Performance Status, symptomatic CN invasion, WHO classification, EBV-DNA, and N classification (Table 7) between the IC group and the non-IC group. Better response to IC may occur in MNI patients and potentially associated with the improved prognoses of MNI patients.

**Table 6:** Adjusted multivariate analysis for IC in T4 patients with or without MR-detected MNI

OS	Variables	HR	95% CI	*P* value
T4 patients with MR-detected MNI	IC	0.35	0.15–0.80	0.014
	Age	1.02	0.99–1.06	0.188
	Symptomatic CN invasion	2.99	1.21–7.37	0.018
	MMI	1.34	0.54–3.29	0.526
T4 patients without MR-detected MNI	IC	0.54	0.14–2.01	0.356
	Age	1.04	1.00–1.08	0.072
	Symptomatic CN invasion	2.14	0.66–6.94	0.203
	MMI	3.60	1.16–11.18	0.027

*OS* overall survival, *HR* hazard ratio, *CI* confidence interval, *CN* cranial nerve, *IC* induction chemotherapy, *MMI* masticatory muscles involvement, *MNI* mandibular nerve involvement

**Table 7:** Chi-square test results for T4 patients with MR-detected MNI who were and were not treated with IC

Variables	IC not administered	IC administered	*P* value
	*n* = 43	*n* = 98	
Age (years)			0.782
13–75	18–74	13–75	
KPS			0.382
80	1 (2.3%)	6 (6.1%)	
90	42 (97.7%)	90 (91.8%)	
100	0 (0%)	2 (2%)	
Symptomatic CN invasion			0.072
No	38 (88.4%)	72 (73.5%)	
Yes	5 (11.6%)	26 (26.5%)	
Histologic type			0.682
II	1 (2.3%)	4 (4.1%)	
III	42 (97.7%)	94 (95.9%)	
EBV load (1000 copies/ml)			1.000
< 1	12 (27.9%)	28 (28.6%)	
< 10	11 (25.6%)	24 (24.5%)	
≥ 10	20 (46.5%)	46 (46.9%)	
N classification			1.000
N0/1	33 (76.7%)	75 (76.5%)	
N2/3	10 (23.3%)	23 (23.5%)	

*CN* cranial nerve, *EBV* Epstein–Barr virus, *IC* induction chemotherapy, *MNI* mandibular nerve involvement, *KPS* Karnofsky Performance Status

## Discussion

In this study, we conducted a retrospective analysis to verify the prognostic value of MR-detected MNI and its significance for IC administration for patients with T4 disease. We found that patients with MR-detected MNI had better 5-year OS comparison to those without MNI in T4 patients, independent of the influence of the invasion of masticatory muscles or cavernous sinus, suggesting that this nerve invasion could be validated as a useful and superior predictor for T4 patients’ prognostication. Furthermore, our results showed that IC use significantly improved the OS of patients with MNI, implying that more satisfactory response to IC may exist in patients with this nerve invasion, especially excluding the influence of tumor burden, clinical characteristics, invasion pattern, and other CN invasion. These findings are of great significance to both radiologists and clinicians.

With the IMRT application and MR examination, the overall control of T4 NPC, however, hasn’t markedly enhanced over the past decades (Lee et al. 2009; Hong et al. 2018). Identifying potential imaging markers associated with survival benefits would allow appropriate treatment strategy tailoring for different patient subgroups, especially in the current anatomy-based staging system. Hu et al. (2011) and Chen et al. (2012), respectively, revealed that tumors with involvement of the unilateral cavernous sinus or the parasellar region only or tumor involvement with masticator space only was associated with better prognosis. A study from Tsung-Min Hung et al. (Hung et al. 2014) showed that the presence of prepontine cistern invasion served as a prognostic factor for high risk of death and distant metastasis in patients with T4 NPC and suggested systematic treatment for these patients. Liao et al. (2016) indicated that patients with cavernous sinus invasion had poor survival outcomes and the use of IC might decrease the mortality and distant micrometastases; however, no significant difference was observed between the group who were treated with IC and the groups who were not. In present study, MR-detected MNI could independently predict better OS of T4 patients and have superior prognostic value to that of masticatory muscles or cavernous sinus invasion, indicating that MR-detected MNI may be a promising imaging marker that could optimize the heterogeneity of T4 patients.

Specifically, we observed that MR-detected MNI had an independent and protective prognostic value for T4 NPC patients. After failing to find the potential variables that distribute differently between the MNI and non-MNI groups, including clinical characteristics, tumor burden, invasion pattern, and other CN invasion, we hypothesized that the protective effect might be associated with better response to treatment in patients with MR-detected MNI. Moreover, we found that IC administration could significantly improve OS in patients with MR-detected MNI, and the prognosis of these patients was approaching that of T3 patients. If without IC administration, there was no significant difference in the OS between the patients with and without MR-detected MNI (Fig. 5). No difference in the frequency of IC use between the MNI and non-MNI groups but a better prognosis was observed in the MNI group after the use of IC in both groups, which may indirectly suggest that nasopharyngeal tumors that tend to invade the mandibular nerve may respond better to IC. Further, IC administration is a routine treatment strategy for advanced patients. It may be of great significance for clinicians to find out who are not sensitive to this approach and change to more intensive treatment in time.

Liu et al. (2009) pointed out that MR-detected CN invasion had an adverse effect on prognostication in locally advanced patients. That MR-detected MNI associated with better OS contradicts the current view that perineural invasion indicates a poor prognosis. Patients with the T4 stage were our main subjects. If not receiving IC, the prognosis in the T4 patients with MR-detected MNI was still poor. The phenomenon that IC administration improved the prognosis of patients with CN invasion has been reported in a few studies. In patients with resectable colorectal liver metastases, perineural invasion lose its value on prognosis after undergoing neoadjuvant chemotherapy (Stift et al. 2018). The mechanism of the perineural invasion shows that multiple factors, such as nerve microenvironment, chemokines, or cellular adhesion molecules, play potential roles in tumor invasion and dissemination, implicating the need for targeted therapeutic management (Liebig et al. 2009). For instance, Scanlon et al. (2015) identified the neuropeptide galanin receptor-induced pathway as a potential treatment target for perineural invasion. Giz et al. (2010) observed that glial cell-derived neurotrophic factor played an important role in dynamic interactions between nerves and cancer cell migration and suggested potential therapy for paracrine regulation against tumor invasion in pancreatic cancer. However, there is no standardization of targeted therapy for nerve invasion. IC for MNI in advanced NPC is an attempt to explore the effective therapeutic management of CN invasion beyond inconsistent and complex mechanisms. We guess that although the mandibular nerve is accompanied by abundant blood vessels and increases the probability of metastasis, this may amplify the advantage of the main effect of IC, that is, to reduce micrometastasis. The relationship between the biological behavior of tumors and their response to IC warranted more evaluation in future studies.

While IC decreased the mortality in T4 patients with MR-detected MNI, it did not significantly improve distant metastasis and local control, whereas distant metastasis remains the primary cause of treatment failure even in the IMRT era (Lee et al. 2014). This may be because of following reasons: (1) the small number of patients with stage T4 led to a failure to separate the survival curves for DMFS and LRFS, while DMFS and LRFS have a similar trend with OS; (2) a different induction regimen might have mainly influenced the efficacy of treatment on OS and PFS, rather than other end points (Hong et al. 2018; Zhang et al. 2019). This may be the reason why the subclassification of patients with T4 had no significant effect on DMFS and LRFS. This finding highlights the prognostic significance of MR-detected MNI and the need for taking this imaging marker into the account when stratifying patients for neoadjuvant chemotherapy.

There are some limitations in this study. First, this was a retrospective study, and we cannot strictly control the heterogeneity of our data. Moreover, we lacked specific MR scan sequences (e.g., three-dimensional turbo spin-echo short inversion time inversion recovery sequence) and functional MR imaging (e.g., diffusion tensor imaging) that might help to properly assess the biological behavior of tumor invasion of the mandibular nerve. Second, NPC is the radiosensitive head and neck malignancy; and the main treatment of NPC is the nonoperative therapeutic management, which leads to the lack of histological confirmation of perineural invasion in NPC. It should be stressed that this study showed no pathologic correlation between MNI and MR findings, but we believe the involvement of mandibular nerve was not unusual and it was possible to assess it accurately using MR findings. The inter-observer variability of assessing MNI was excellent in our study. Last, only a small number of patients were included in the analysis. More data from prospective, multicenter studies is warranted in the future.

In conclusion, the presence of MR-detected MNI suggested better OS in T4 patients and may be a potential imaging marker that guides clinicians’ decision-making. Our results may facilitate treatment strategies and prognosis in T4 patients with NPC.

## Data Availability

The data that support the findings of this study are available from the corresponding author upon request.
